# Factors Associated with Self-Reported Voice Change in the Hospitalized Burn Population: A Burn Model System National Database Study

**DOI:** 10.3390/ebj5020010

**Published:** 2024-04-30

**Authors:** Kaitlyn L. Chacon, Edward Santos, Kara McMullen, Lauren J. Shepler, Carla Tierney-Hendricks, Audra T. Clark, Chiaka Akarichi, Haig A. Yenikomshian, Caitlin M. Orton, Colleen M. Ryan, Jeffrey C. Schneider

**Affiliations:** 1 Department of Physical Medicine and Rehabilitation, Schoen Adams Research Institute, Harvard Medical School, Spaulding Rehabilitation Hospital, Boston, MA 02129, USA; klchacon@mgb.org (K.L.C.); esantos16@mgb.org (E.S.); lshepler@mgb.org (L.J.S.); chendricks@mghihp.edu (C.T.-H.); 2 Department of Rehabilitation Medicine, University of Washington, Seattle, WA 98104, USA; mcmulk@uw.edu; 3 Department of Surgery, University of Texas Southwestern Medical Center, Dallas, TX 75390, USA; audra.clark@utsouthwestern.edu (A.T.C.); chiaka.akarichi@utsouthwestern.edu (C.A.); 4 Division of Plastic Surgery, Keck School of Medicine, University of Southern California, Los Angeles, CA 90033, USA; 5 Department of Surgery, UW Medicine Regional Burn Center, Harborview Medical Center, University of Washington, Seattle, WA 98104, USA; corton@uw.edu; 6 Department of Surgery, Massachusetts General Hospital, Harvard Medical School, Shriners Hospitals for Children, Boston, MA 02114, USA; cryan@mgh.harvard.edu

**Keywords:** voice change, mechanical ventilation, tracheostomy, dysphonia

## Abstract

Voice plays a prominent role in verbal communication and social interactions. Acute burn care often includes intubation, mechanical ventilation, and tracheostomy, which could potentially impact voice quality. However, the issue of long-term dysphonia remains underexplored. This study investigates long-term self-reported voice changes in individuals with burn injuries, focusing on the impact of acute burn care interventions. Analyzing data from a multicenter longitudinal database (2015–2023), self-reported vocal changes were examined at discharge and 6, 12, 24, and 60 months after injury. Out of 582 participants, 65 reported voice changes at 12 months. Changes were prevalent at discharge (16.4%) and persisted over 60 months (11.6–12.7%). Factors associated with voice changes included flame burn, inhalation injury, tracheostomy, outpatient speech-language pathology, head/neck burn, larger burn size, mechanical ventilation, and more ventilator days (*p* < 0.001). For those on a ventilator more than 21 days, 48.7% experience voice changes at 12 months and 83.3% had received a tracheostomy. The regression analysis demonstrates that individuals that were placed on a ventilator and received a tracheostomy were more likely to report a voice change at 12 months. This study emphasizes the need to understand the long-term voice effects of intubation and tracheostomy in burn care.

## 1. Introduction

Voice, from the Latin word vocem, plays a large role in verbal communication and social interactions [1,2]. The voice is the channel through which ideas, emotions, and personalities are communicated [2]. When injuries to the head and neck occur, patients may be left with vocal changes that can impact their communication and quality of lifelong term [3]. Although burn injury has a 96.8% survival rate, many survivors face long-term challenges [4,5,6]. Some of these challenges include physical and mental health deficits, impacting life satisfaction. Dysphonia, an impairment in voice, and dysphagia, a swallowing disorder, can persist through the acute stage of burn injury and manifest as chronic issues, especially in individuals who also have had an inhalation injury [6,7]. 

Studies have shown that some burn injuries result in long-term damage to laryngeal and pharyngeal structures [8,9,10,11]. Burns due to direct thermal contact caused by hot liquids, flames, or steam can directly damage the larynx and pharynx [12]. Likewise, the inhalation of superheated gases and toxic substances during a fire can lead to damage of the upper airway [13]. Delayed complications from burn injuries, such as infection and scarring, may also affect the larynx and pharynx [14]. Scar tissue formation (fibrosis) can cause long-term constriction or narrowing of the airway [14]. 

A systematic review noted that dysphagia and dysphonia were the most prevalent sequelae of patients diagnosed with laryngeal injuries [11]. Other studies have reported a decline in voice quality and some degree of hoarseness after burn and inhalation injury [9]. Early management plans for burn patients often include intubation, which may impact voice quality as well [8,15]. Airway swelling from burns affecting the face, neck, or upper chest can compromise breathing, making intubation necessary to maintain a clear airway, especially in cases of inhalation injury associated with extensive burns [16]. Additionally, intubation allows for mechanical ventilation, aiding oxygenation, and ventilation support [17]. The length of intubation and number of laryngeal procedures is associated with long-term laryngeal function [9,18,19,20,21]. 

Dysphagia and laryngeal status after a burn injury has been well documented, while the issue of long-term dysphonia remains underexplored. Thus, this study aims to describe the frequency with which burn survivors report vocal changes up to five years after injury and examine factors associated with voice changes.

## 2. Materials and Methods

### 2.1. Burn Model System National Database

This study used data from the Burn Model System (BMS) National Database, funded by the National Institute on Disability, Independent Living, and Rehabilitation Research. The BMS National Database was established in 1994 to explore the long-term physical and psychosocial outcomes of burn survivors and is one of the world’s most extensive longitudinal datasets of burn injury outcomes. Informed consent is obtained from all participants, and each BMS site’s Institutional Review Board oversees data collection. The sites included in this study were the Boston-Harvard Burn Injury Model System (BH-BIMS), Boston, MA, USA; North Texas Burn Rehabilitation Model System, Dallas, TX, USA; Northwest Regional Burn Model System, Seattle, WA, USA; and the University of Texas Medical Branch/Shriners Hospital Pediatric Burn Model System Galveston, TX, USA.

Data from participants who were burned between 2015 and 2023, age greater than or equal to 18 years at the time of injury, alive at discharge, and consented to participate in the BMS National Database were included in this study. Current criteria for inclusion in the BMS National Database are those who require autografting or amputation surgery for wound closure and meet one of the following criteria: (1) 0–64 years of age with a burn injury ≥20% total body surface area (TBSA), (2) ≥65 years of age with a burn injury ≥10% TBSA, (3) any age with a burn injury to their face/neck, hands, or feet, (4) any age with a high-voltage electrical burn injury. The BMS database enrollment criteria have been modified over time, and these changes are detailed on the BMS National Data and Statistical Center’s website: https://burndata.washington.edu/ (accessed on 30 January 2023) [22,23].

### 2.2. Demographic and Clinical Characteristics

The following demographic and clinical characteristics were collected by medical record abstraction or self-report: age, race, Hispanic/Latino ethnicity, sex, education, employment, BMS site, alcohol and drug misuse history (both variables from the Cut down, Annoyed, Guilty, and Eye-opener (CAGE) Scale; the CAGE Scale is considered positive if the score is greater than or equal to 2; pre-injury recall collected at discharge) [24,25], burn size (percent total body surface area burned, TBSA), ventilator days (>1 day), burn etiology, inhalation injury (Y/N), tracheostomy (Y/N), outpatient speech-language pathology services (Y/N), head/neck burn, multiple trips to the operating room (≥2; Y/N), and mechanical ventilation. Alcohol and drug misuse were included because they have been shown to affect the vocal cords, potentially leading to inflammation, dehydration, and changes in vocal fold function [26,27,28]. 

### 2.3. Voice Outcomes

The primary outcome was the item, “Do you currently have change in voice?” (dichotomous response options: Y/N) in the Adult Review of Systems section of the BMS follow-up questionnaire at the 12-month time point. This item is also collected at discharge and at 6, 24, and 60 months after burn injury. The 12-month time point was chosen a priori by the authors because it is a sufficient time since injury to capture long-term voice outcomes; additionally, this time point ensured a more robust sample size given documented higher participant attrition rates at more distal time points [29].

### 2.4. Data Analysis

Demographic and clinical characteristics were summarized and compared for participants with and without voice change at 12 months. They were compared using non-parametric tests (Wilcoxon–Mann–Whitney) due to the non-normality of data for continuous variables and chi-square tests or Fisher’s exact (depending on sample size) for categorical variables; significance level was adjusted for multiple comparisons using Bonferroni’s method to *p* < 0.003. The frequency of voice change was examined at all follow-up time points: discharge, six months, 12 months, 24 months, and 60 months after burn injury. In addition, a subgroup analysis examined participants with repeated outcome measurements to examine the percentage of participants reporting voice change at discharge and at each follow-up time point (6, 12, 24, and 60 months). 

An omnibus (chi-square) test was used to examine if there were statistically significant differences in the percent of burn survivors experiencing voice change by ventilator days (categories: 0, 1–10, 11–20, 21+ days). Likewise, the percentage of tracheostomies by ventilator days was at 12 months compared for those with and without a voice change. Tracheostomies are often considered after prolonged endotracheal intubation exceeding 21 days. Thus, different ventilator day durations were used to compare the percentage of those with a tracheostomy to those with voice change [30]. 

A logistic regression model assessed the association between voice change and demographic and clinical variables (age, sex, BMS site, burn size, etiology, inhalation injury, tracheostomy, ventilator (Y/N), burn location, and multiple operations) at 12 months, which was the primary outcome of this study. Robust standard errors accounted for heteroskedasticity, and the significance value was set to 0.05. Two additional analogous exploratory logistic regression models were conducted to assess the association between voice change at 24 and 60 months with demographic and clinical variables (age, sex, BMS site, burn size, etiology, inhalation injury, tracheostomy, ventilator (Y/N), burn location, and multiple operations). In addition, race, ethnicity, education, employment, history of alcohol and drug use (CAGE scale) were not significantly different between groups in the descriptive analysis thus, they were not included the regression model.

## 3. Results

### 3.1. Study Sample and Comparison of Characteristics between Groups

This study included 582 persons with a burn injury (65 self-reported a change in voice and 517 reported no change in voice at 12 months). The group with reported voice changes at 12 months had larger burn size (31.1% vs. 15.5% TBSA; *p* < 0.001) and greater ventilator days (25.9 ± 25.6 vs. 12.9 ± 21.2 days, *p* < 0.001). Additionally, the group with voice changes were more likely to have a flame burn etiology (83.1% vs. 52.7%, *p* < 0.001), inhalation injury (32.8% vs. 8.7%, *p* < 0.001), tracheostomy (29% vs. 4.3%, *p* < 0.001), outpatient speech-language pathology services (7.7% vs. 1%, *p* < 0.001), head/neck burn (78.5% vs. 45.4%, *p* < 0.001), multiple trips to the operating room (76.9% vs. 48.7%, *p* < 0.001), and require mechanical ventilation (67.2% vs. 19.7%, *p* < 0.001). Age, race, ethnicity, sex, education, employment, BMS site, and alcohol and drug misuse history were not statistically different between groups. A comparison of demographic and clinical characteristics between groups is presented in Table 1.

### 3.2. Self-Reported Voice Outcomes over Time

Self-report change in voice was most commonly reported at discharge (16.4%) and persisted over the follow-up time points (11.2–12.7%). The information presented in Table 2 is cross-sectional, resulting in varying sample sizes at each time point. Additionally, it excludes individuals whose data collection periods had not commenced (for instance, the 60-month data point excludes participants who sustained burn injuries after 2019).

The frequency of voice changes for participants with repeated measures at discharge and follow-up time points is summarized in Table 3. Participants with repeated measures reported change in voice at discharge at 15.8–19.6% and change in voice at follow-up time points ranged from 10.7% to 17.1%.

### 3.3. Association of Voice Changes with Ventilator Days and Tracheostomy

**Figure 1:**
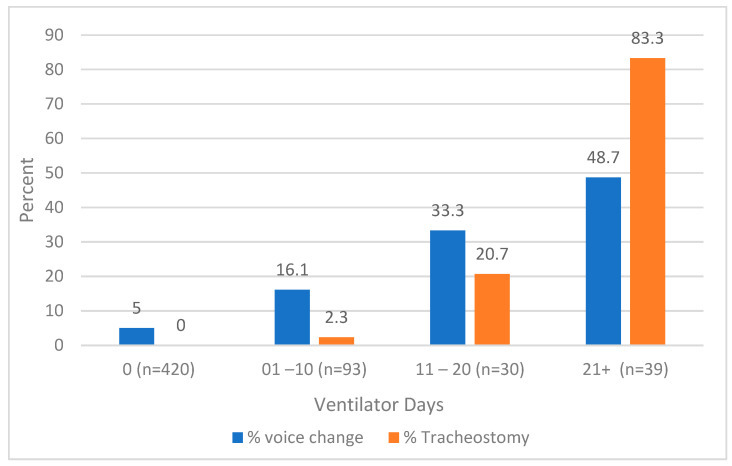
Histogram of the association between voice change and tracheostomy status by ventilator days. Voice change and tracheostomy status are associated with ventilator days at 12 months (*p* < 0.001). Differences were examined using the omnibus (chi-square) test.

At 12 months, self-reported changes in voice were observed to increase with more ventilator days. For participants with 21+ days on a ventilator, 48.7% reported a change in voice at 12 months. Similarly, the percentage of tracheostomies increased with more ventilator days. For participants with 21+ days on a ventilator, 83.3% had a tracheostomy. The relationship between voice change, tracheostomy status and ventilator days at 12 months is illustrated in Figure 1.

### 3.4. Factors Associated with Voice Change 

The logistic regression analysis examined the association between self-reported voice changes at 12 months and demographic and clinical factors (Table 4). Results showed that participants with a tracheostomy were 184% (*p* = 0.027) more likely to report change in voice at 12 months, and those participants on a ventilator were 331% (*p* < 0.001) more likely to report change in voice when controlling for demographic and other clinical factors. Additionally, an exploratory logistic regression investigated the correlation between self-reported voice changes at 24 months and various demographic and clinical factors. It demonstrated that age (OR: 1.05, *p* < 0.001) and tracheostomy (OR: 39.75, *p* < 0.001) increased the odds of reporting a voice change. Another exploratory regression model was not conducted for the 60-month time point due to the small sample size for analysis.

## 4. Discussion

Self-reported voice changes are common among people experiencing burn injuries and can persist for up to five years after injury [31]. This study found that approximately one in six participants reported voice changes at acute hospital discharge, and one in nine reported changes up to five years after injury. In addition, those who were treated with a ventilator and received a tracheostomy showed a higher likelihood of reporting voice changes at one year. These findings may inform the longitudinal care of burn patients regarding voice issues and a growing understanding of the long-term implications of acute interventions, such as mechanical ventilation and tracheostomy. 

This study provides evidence that dysphonia is a long-term complication, which contributes to understanding burns as a chronic condition [6]. Other research supports that dysphonia ‘s chronicity can affect all ages and genders with a higher prevalence in patients who use their voice more frequently due to work-related responsibilities [7,32,33]. Additionally, dysphonia negatively impacts quality of life, including social interactions, work performance, and pain [34]. A scoping review examining the long-term laryngotracheal complications after inhalation injury noted that dysphonia was a frequent sequela after injury [35]. Close monitoring and timely interventions to address dysphonia are important. The authors suggest providers include questions about voice during the follow-up evaluations to screen for dysphonia. For symptomatic patients, referrals to otolaryngologists for further evaluation and speech language pathologists for voice rehabilitation should be considered. A possible area for further exploration involves developing long-term care models that address prevalent changes in vocal quality among people living with burn injuries along with patient-reported outcome measures that capture the impact of voice changes on daily tasks. 

At 12 months after injury, individuals living with burn injury exhibited higher odds of reporting a change in voice if they were on a ventilator and had received a tracheostomy. Studies have shown that prolonged intubation can exacerbate the risk of vocal cord injury, thus emphasizing the potential benefits of minimizing the duration of intubation to mitigate voice quality complications [16]. Over the last two decades, many have debated the proper timing of tracheostomy for patients with prolonged mechanical ventilation [16,18,35]. The American College of Chest Physicians guidelines recommend considering a tracheostomy in patients receiving mechanical ventilation for more than 21 days [36,37]. The data from this study provide additional detail on the long-term implications of prolonged mechanical ventilation and tracheostomy as this practice was associated with increased odds of experiencing voice changes 12 months after injury. 

There are multiple limitations in this study. There was less voice change data at more distal time points due to loss to follow-up, and some participants had not yet reached that follow-up period. Nonetheless, patterns regarding the frequency of self-reported voice changes emerged by examining all data cross-sectionally (Table 2) and cohorts with repeated measures (Table 4). Given that the BMS database inclusion criteria select those with more severe burn injuries, this introduces selection bias; additionally, follow-up data may be affected by response bias [36]. However, prior work has noted that the BMS database is representative of the national burn population [38]. In this study, there are likely confounding factors involved in the inhalation injury, intubation, and tracheostomy data contributing to these results. The BMS database does not include days from tracheostomy to decannulation or duration of overlap between tracheostomy and ventilation. Therefore, the dataset is not able to assess if duration of tracheostomy or overlap in tracheostomy and ventilator is related to subsequent dysphonia. Thus, this study cannot conclusively assess if the timing of transitioning from endotracheal intubation to tracheostomy impacts voice outcomes. A study comparing dysphonia and dysphagia outcomes of early tracheostomy (at 14 days) versus current guidelines (21 days) would be an important future study.

It is important to note that the voice change data in this dataset were based on participant self-report without formal speech therapy assessment or validated voice outcome measures, and it also lacks information on the nature and frequency of voice interventions received by burn survivors, potentially affecting the course of their voice symptoms. It is possible that some burn survivors experienced voice changes as a result of other health issues unrelated to their burn injury. To address these limitations, future studies may utilize objective standardized measures of voice function and anatomy with Speech-Language Pathology and Otolaryngology assessments to capture the severity and physiology of vocal impairments. Despite these limitations of the dataset and study design, this study is one of the first to examine long term self-reported dysphonia outcomes after burns in a multicenter database and therefore is a starting point in better understanding these outcomes.

## 5. Conclusions

The study found that self-reported voice changes are common and often persist for up to five years in the hospitalized burn population. Notably, factors such as mechanical ventilation and tracheostomy are associated with persistent voice changes after injury. Nonetheless, the findings of this study should be considered in the context of its limitations, which notably included self-reported voice changes without comprehensive speech therapy or otolaryngology assessments. Future studies should examine voice outcomes utilizing standardized assessments as well as the impact of speech-language pathology and other interventions on those with long-term dysphonia after burns. 

## Figures and Tables

**Table 1:** Comparison of demographic and clinical variables between participants with and without voice changes at 12 months (*n* = 582).

Variable	With Voice Change (*n* = 65)	Without Voice Change (*n* = 517)	*p*-Value
Age, mean years (SD)	48.2 (16.3)	46.8 (16.2)	0.59
Male, n (%)	40 (61.5)	359 (69.4)	0.20
Hispanic/Latino ethnicity, n (%)	16 (25.0)	100 (20.1)	0.36
Race, n (%)			
White	55 (87.3)	416 (83.7)	0.21
African American/Black	3 (4.8)	48 (9.7)	
Other ^1^	5 (7.9)	33 (6.6)	
Education, n greater than high school (%)	25 (42.4)	254 (56.3)	0.04
Burn Model System site, n (%)			
Site A	20 (30.8)	188 (36.4)	0.614
Site B	21 (32.3)	174 (33.7)	
Site D	24 (36.9)	152 (29.4)	
Employed at time of injury, n (%)	36 (75.0)	333 (80.6)	0.36
Burn size, mean percent TBSA (SD)	31.1 (24.2)	15.5 (16.8)	<0.001 *
Ventilator days, mean (SD) ^2^	25.9 (25.6)	12.9 (21.2)	<0.001 *
Mechanical ventilation, n (%)	43 (67.2)	98 (19.7)	<0.001 *
Alcohol misuse history, n (%)	6 (10.0)	64 (13.4)	0.46
Drug misuse history, n (%)	4 (6.8)	20 (4.3)	0.39
Burn etiology, n (%)			
Fire/Flame	54 (83.1)	272 (52.7)	<0.001 *
Other ^3^	11 (16.9)	244 (47.3)	
Inhalation injury, n (%)	21 (32.8)	45 (8.7)	<0.001 *
Tracheostomy, n (%)	18 (29.0)	20 (4.3)	<0.001 *
Outpatient speech services at 12 months, n (%)	5(7.7)	5 (1.0)	<0.001 *
Head/neck burn, n (%)	51 (78.5)	234 (45.4)	<0.001 *
Multiple trips to the operating room, n (%) ^4^	50 (76.9)	252 (48.7)	<0.001 *

Differences between groups was assessed using Wilcoxon–Mann–Whitney for continuous variables and chi-square tests for Fisher’s exact for categorical variables. Significance level has been adjusted for multiple comparisons using Bonferroni’s method to 0.003 and designated with *. ^1^ “Other” race includes Asian, American Indian/Alaskan Native, Native Hawaiian/Other Pacific Islander, more than one race, some other race and unknown race. ^2^ Ventilator days variable includes participants that had 1 or more days on the ventilator (with voice change *n* = 43, without voice change *n* = 115). ^3^ “Other” Burn etiology includes scald, contact with hot object, grease, tar, chemical, hydrofluoric acid, radiation, UV light. ^4^ Multiple trips to operating room defined as two or more during acute hospitalization. TBSA = total body surface area.

**Table 2:** Self-reported change in voice at each follow-up time point after burn injury.

Time Point	Sample Size, n	Change in Voice, *n* (%)	Missing Data
Discharge	898	147 (16.4)	174
6 months	670	78 (11.6)	501
12 months	582	65 (11.2)	516
24 months	499	56 (11.2)	463
60 months	157	20 (12.7)	554

**Table 3:** Frequency of “change in voice” at discharge and each follow-up among those with repeated measures.

Participants Who Responded to “Change in Voice” Item at Discharge and Follow-Up, *n* (Follow-Up Time Point)	Voice Change at Discharge, *n* (%)	Voice Change at Follow-Up, *n* (%)
589 (6 months)	93 (15.8)	67 (11.4)
481 (12 months)	82 (17.1)	56 (11.6)
338 (24 months)	63 (18.6)	36 (10.7)
51 (60 months)	10 (19.6)	9 (17.7)

**Table 4:** Logistic regression analysis examining associations between self-reported changes in voice at 12-months and demographics and clinical characteristics.

Variable	Odds Ratio	Robust SE	Z	*p*-Value	95% Cl
Age	1.02	0.01	1.93	0.054	0.99–1.04
Female	1.87	0.61	1.92	0.055	0.99–3.54
Burn Model System site					
Site B	1.15	0.47	0.36	0.722	0.53–2.50
Site D	1.34	0.54	0.72	0.472	0.61–2.94
Burn size, TBSA	1.00	0.01	0.3	0.767	0.98–1.02
Etiology of burn					
High-voltage electrical	0.49	0.39	−0.9	0.367	0.10–2.31
Other ^1^	0.46	0.19	−1.83	0.067	0.20–1.06
Inhalation injury	1.01	0.41	0.03	0.976	0.45–2.30
Tracheostomy	2.84	1.34	2.21	0.027	1.13–7.14
Ventilator	4.31	1.71	3.69	<0.001	1.98–9.37
Head/neck burn	0.60	0.23	−1.35	0.177	0.28–1.26
Multiple operations	1.01	0.4	0.04	0.971	0.47–2.18

A logistic regression model fit with the outcome of voice change (1 = yes, 0 = no) at twelve months and the dependent variables being age, sex, site, TBSA burn size, etiology of injury, inhalation injury, tracheostomy, ventilator (yes/no), head/neck burn, and multiple trips to the OR. Reference category is Site A for BMS site, flame for etiology of burn, multiple operations defined as two or more during acute hospitalization; ^1^ “Other” burn etiology includes scald, contact with hot object, grease, tar, chemical, hydrofluoric acid, radiation, UV light.; Significance level is (*p* < 0.05). TBSA = total body surface area.

## Data Availability

The Burn Injury Model System National Database is a prospective, longitudinal, multi-center research data repository that contains measures of functional and psychosocial outcomes following burns. The data are free and publicly available at https://burndata.washington.edu/ (accessed on 30 January2023).
